# Deciphering the shared mechanisms of Gegen Qinlian Decoction in treating type 2 diabetes and ulcerative colitis via bioinformatics and machine learning

**DOI:** 10.3389/fmed.2024.1406149

**Published:** 2024-06-19

**Authors:** Faquan Hu, Liyuan Xiong, Zhengpin Li, Lingxiu Li, Li Wang, Xinheng Wang, Xuemei Zhou, Yujiao Zheng

**Affiliations:** College of Traditional Chinese Medicine, Anhui University of Chinese Medicine, Hefei, China

**Keywords:** Gegen Qinlian Decoction, type 2 diabetes, ulcerative colitis, bioinformatics, network pharmacology, traditional Chinese medicine

## Abstract

**Background:**

Although previous clinical studies and animal experiments have demonstrated the efficacy of Gegen Qinlian Decoction (GQD) in treating Type 2 Diabetes Mellitus (T2DM) and Ulcerative Colitis (UC), the underlying mechanisms of its therapeutic effects remain elusive.

**Purpose:**

This study aims to investigate the shared pathogenic mechanisms between T2DM and UC and elucidate the mechanisms through which GQD modulates these diseases using bioinformatics approaches.

**Methods:**

Data for this study were sourced from the Gene Expression Omnibus (GEO) database. Targets of GQD were identified using PharmMapper and SwissTargetPrediction, while targets associated with T2DM and UC were compiled from the DrugBank, GeneCards, Therapeutic Target Database (TTD), DisGeNET databases, and differentially expressed genes (DEGs). Our analysis encompassed six approaches: weighted gene co-expression network analysis (WGCNA), immune infiltration analysis, single-cell sequencing analysis, machine learning, DEG analysis, and network pharmacology.

**Results:**

Through GO and KEGG analysis of weighted gene co-expression network analysis (WGCNA) modular genes and DEGs intersection, we found that the co-morbidity between T2DM and UC is primarily associated with immune-inflammatory pathways, including IL-17, TNF, chemokine, and toll-like receptor signaling pathways. Immune infiltration analysis supported these findings. Three distinct machine learning studies identified IGFBP3 as a biomarker for GQD in treating T2DM, while BACE2, EPHB4, and EPHA2 emerged as biomarkers for GQD in UC treatment. Network pharmacology revealed that GQD treatment for T2DM and UC mainly targets immune-inflammatory pathways like Toll-like receptor, IL-17, TNF, MAPK, and PI3K-Akt signaling pathways.

**Conclusion:**

This study provides insights into the shared pathogenesis of T2DM and UC and clarifies the regulatory mechanisms of GQD on these conditions. It also proposes novel targets and therapeutic strategies for individuals suffering from T2DM and UC.

## 1 Introduction

Emerging research posits that chronic tissue inflammation is a central player in the pathogenesis of Type 2 Diabetes Mellitus (T2DM), characterized by a state of low-grade inflammation (1). The disturbance in gut mucosal ecology in individuals with T2DM, combined with the active migration of intestinal flora to mesenteric adipose tissue (MAT) and the bloodstream, results in a continuous influx of inflammatory antigens (2). Ulcerative colitis (UC) is a chronic, nonspecific inflammatory condition characterized by extensive mucosal inflammation in the colon (3), typically arising from an imbalance between the gut flora and the immune system (4). Although T2DM and UC share common features, such as disruptions in gut microbiota and intestinal mucosa inflammation, the precise mechanisms underlying their co-occurrence remain unclear.

Currently, there is no cure for Type 2 diabetes. Despite the recent successful development of numerous antidiabetic drugs, single-target treatments are increasingly seen as inadequate due to individual variations, diverse pathogenesis, and issues related to drug and body resistance (5). Similarly, ulcerative colitis carries a heightened risk of adverse events, treatment resistance, and loss of response over time, highlighting the limitations of current therapies (6). Thus, multi-target drugs offer greater potential advantages over single-target drugs, underscoring the need to continually identify new targets to develop effective and safe therapies. Traditional Chinese medicine formulations are characterized by their multi-component approach, targeting multiple pathways and targets simultaneously.

The venerable Chinese herbal prescription, Gegen Qinlian Decoction (GQD), traces its origins to the era of the Eastern Han Dynasty. This formulation, consisting of four vital herbs—*Radix puerariae*, *Radix scutellariae, Rhizoma coptidis*, and *Glycyrrhizae Radix,* represents a traditional prescription deeply rooted in the principles of Traditional Chinese Medicine (TCM), specifically tailored for addressing intestinal damp-heat syndrome. A large-scale randomized controlled study (RCT) has demonstrated GQD’s efficacy in significantly lowering HbA1c and fasting blood glucose (FBG) levels, offering relief in cases of T2DM (7). Animal experiments suggest that GQD may mitigate systemic and local inflammation by promoting the enrichment of butyrate-producing intestinal flora, thereby ameliorating clinical manifestations associated with T2DM (8). Meta-analyses have shown that GQD effectively alleviates symptoms in individuals with UC, resulting in decreased Ulcerative Colitis Endoscopic Index of Severity (UCEIS) scores and maintaining a low recurrence rate, all while exhibiting minimal adverse events (9). Furthermore, additional animal studies have elucidated the mechanisms underlying GQD’s therapeutic effects in alleviating ulcerative colitis, including the reduction of inflammation and oxidative stress, inhibition of the IL-6/JAK2/STAT3 signaling pathway, restoration of the balance between Treg and Th17 cells in colonic tissues, and enhancement of intestinal barrier function (10, 11).

**Figure 1:**
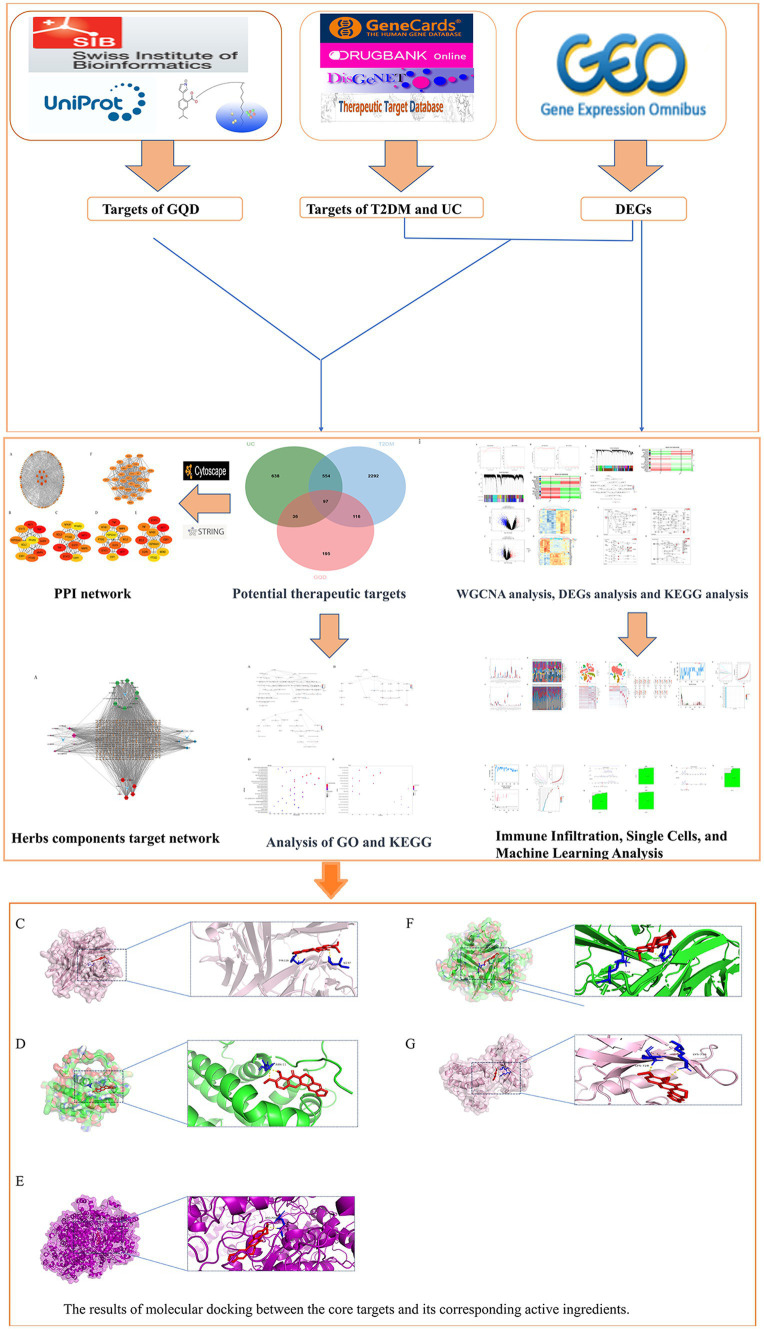
Workflow diagram illustrating the research strategy, encompassing five main components: database preparation, exploration of co-morbidity mechanisms in T2DM and UC, biomarker prediction for GQD treatment, and network pharmacology along with molecular docking analyses.

As bioinformatics advances and the widespread adoption of gene chips continue, their integration into the biomedical domain has become indispensable. The analysis of microarray data emerges as a transformative tool, offering fresh insights into the shared etiological underpinnings of both T2DM and UC. In this investigation, a comprehensive strategy merging bioinformatics and machine learning was employed, drawing upon datasets from the GEO database to unravel the intertwined comorbid mechanisms associated with T2DM and UC. Furthermore, our study delved into network pharmacology, shedding light on the intricate mechanisms governing the utilization of GQD across diverse diseases sharing a common treatment modality (Figure 1).

## 2 Methods

### 2.1 Datasets

We queried the GEO database^1^ to retrieve gene expression profiles of individuals diagnosed with Type 2 Diabetes Mellitus (T2DM) and Ulcerative Colitis (UC), using search terms such as “type 2 diabetes” and “ulcerative colitis.” For the subsequent phase of our investigation, we selected the following GEO datasets: GSE3365, GSE48958, GSE75214, GSE231993, GSE20966, GSE25724, GSE29221, and GSE220939 (Table 1).

**Table 1:** Data sets and their characteristics.

Dataset	Database	Platform	Sample
GSE3365	GEO	GPL96	26 cases of UC and 42 controls
GSE48958	GEO	GPL6244	13 cases of UC and 8 controls
GSE75214	GEO	GPL6244	97 cases of UC and 11 controls
GSE231993	GEO	GPL18573	4 cases of UC and 4 controls
GSE20966	GEO	GPL1352	10 cases of T2DM and 10 controls
GSE25724	GEO	GPL96	6 cases of T2DM and 7 controls
GSE29221	GEO	GPL6947	3 cases of T2DM and 3 controls
GSE220939	GEO	GPL11154	16 cases of T2DM and 6 controls

### 2.2 Construction of weighted gene co-expression networks

We leveraged Weighted Gene Co-Expression Network Analysis (WGCNA) to pinpoint clusters of closely linked genes. Applying WGCNA, we scrutinized the differential genes within the GEO datasets GSE20966 and GSE75214, unveiling co-expression modules and pivotal genes intricately linked to both T2DM and UC. In the dataset about T2DM, the outlier GSM524165 was omitted, and subsequent parameter selection involved R2 = 0.85 and β = 9. In the case of the UC dataset, parameters R2 = 0.85 and β = 8 were applied. The matrices undergo sequential transformation and derivation, ultimately yielding the Topological Overlap Matrix (TOM). Hierarchical clustering, with a minimum module size set at 30, is employed to identify modules. Subsequently, feature genes are computed, and hierarchical clustering is applied to the modules. Ultimately, we identified the common genes within the top three significantly ranked modules associated with both T2DM and UC. Subsequently, we subjected these intersecting genes to a thorough GO enrichment analysis.

### 2.3 Acquisition of differentially expressed genes (DEGs)

The study analyzed the DEGs using the GSE25724 and GSE48958 datasets. The initial gene expression data undergoes thorough cleaning, with the Robust Multi-array Average (RMA) method employed to equalize sample differences. Subsequently, gene name normalization is necessary to eliminate empty columns and duplicate values, ensuring the acquisition of normalized expression data. Our analysis utilized the R limma package, employing a filtering criterion for DEGs set at |logFC| ≥ 1 and a *p*-value threshold of <0.05. We proceeded to identify the common genes between the two sets of DEGs and subjected this intersection to KEGG pathway enrichment analysis.

### 2.4 Immune infiltration analysis

To precisely evaluate the composition of immune cells in UC compared to T2DM, we conducted calculations utilizing the CIBERSORT algorithm. We utilized the authentic CIBERSORT gene signature file that delineates 22 immune cell subtypes for analyzing the T2DM versus UC dataset. A significance level of *p* < 0.05 denotes a meaningful difference. The datasets utilized were GSE3365 and GSE29221.

### 2.5 Network pharmacology analysis

#### 2.5.1 Acquisition of GQD active ingredients and target proteins

UPLC-Q-TOF/MS analysis revealed a comprehensive profile of 130 active chemical components, out of which 37 components met the criteria of Oral Bioavailability (OB) ≥ 30% and Drug-Likeness (DL) ≥ 0.18 (12). Retrieve the 2D structure of the compound from the PubChem database.^2^ Chemical targets were then predicted using PharmMapper and SwissTargetPrediction (13).

#### 2.5.2 Predictive hub genes for GQD treatment

We employed three machine learning algorithms to systematically screen biomarker targets associated with GQDs for the treatment of T2DM and UC, respectively. LASSO logistic regression selectively assigns coefficients to significant variables by imposing an L1 penalty to remove less relevant ones, thus optimizing the classification model. SVM-RFE analysis, a supervised learning approach, identifies key genes by iteratively eliminating feature vectors derived from SVM. Random forest analysis, rooted in decision trees, evaluates variable importance by scoring each variable (14). The seed was set to 123 for consistency in the analysis. The targets of GQD were compared with the DEGs of T2DM and modules from WGCNA that exhibited a positive correlation with T2DM at *p* < 0.05. The overlapping elements from these three sets were analyzed, and a parallel approach was applied to UC. To enhance diagnostic accuracy and prediction capabilities, diagnostic nomograms were created utilizing hub genes as the foundation.

#### 2.5.3 Common targets of GQD for the treatment of T2DM and UC

Disease-associated targets were queried in the DrugBank,^3^ GeneCards,^4^ TTD,^5^ and DisGeNET^6^ databases using the keywords “Type 2 diabetes” and “Ulcerative colitis” (15, 16). Targets occurring in at least two instances across DrugBank, GeneCards, TTD, DisGeNET, and DEGs datasets are identified as disease targets. The ultimate selection comprises overlapping genes from the targets associated with T2DM, UC, and GQD, serving as potential common targets for GQD treatment of both T2DM and UC.

#### 2.5.4 Protein–protein interaction (PPI) network

TSV files of PPI were obtained by uploading potential therapeutic target genes into the STRING database and constructing networks in Cytoscape 3.9.1. We employed the cytoHubba plugin and computed parameters such as Degree Centrality (DC), Betweenness Centrality (BC), Closeness Centrality (CC), and Maximal Clique Centrality (MCC). Central genes were identified through two methods: firstly, by calculating the top ten targets ranked by each of the four parameters and then determining the overlap among them; secondly, by utilizing the MCODE plugin for cluster analysis, generating a highly connected sub-network.

#### 2.5.5 The analysis of GO and KEGG

To comprehend the shared physiological mechanisms of GQD for both T2DM and UC, we conducted GO and KEGG enrichment analyses of therapeutic targets using the R language. Significance thresholds were set at *p* ≤ 0.05 and *q* ≤ 0.01, and the outcomes were visually presented for comprehensive understanding.

#### 2.5.6 Molecular docking

Key genes were selected from the PPI sub-network, and core chemicals were screened from the drug target network map for subsequent molecular docking analysis. The structures of the core active ingredients were sourced from online databases. Protein stereo structures were also retrieved from databases and subjected to dehydration, hydrogenation, and removal of impurity ligands using PYMOL software. Following that, molecular docking analysis was conducted using Autodock, and the results were graphically presented.

### 2.6 Single-cell sequencing analysis

Seurat objects were initialized by loading gene expression data from the GEO database via the read10X function. Cell curation involved preserving those with a gene count between >200 and < 10,000, while filtering out those with mitochondrial and ribosomal gene proportions exceeding 20%. Following this, standardization and normalization procedures were applied for data uniformity. Spatial relationships between clusters were evaluated using the tSNE method, and subsequent cluster annotations were conducted using the celldex package. The reclassification of cell subpopulations was accomplished through the singleR annotation tool, concurrent with referencing the Thermofisher website to identify genes characterizing different immune cell types. Following observation of the expression patterns of these genes within the clustering results, a manual classification of immune cell classes was performed for annotation purposes. Finally, we have successfully visualized the expression patterns of GQD targets at the single-cell level and elucidated the distribution of the seven core targets.

### 2.7 Statistical analysis

The R packages utilized in this study include WGCNA, GEOquery, reshape2, ggfortify, limma, pheatmap, ggplot2, org.Hs.eg.db, pathview, topGO, and Rgraphviz.

## 3 Results

### 3.1 Construction of WGCNA network

**Figure 2:**
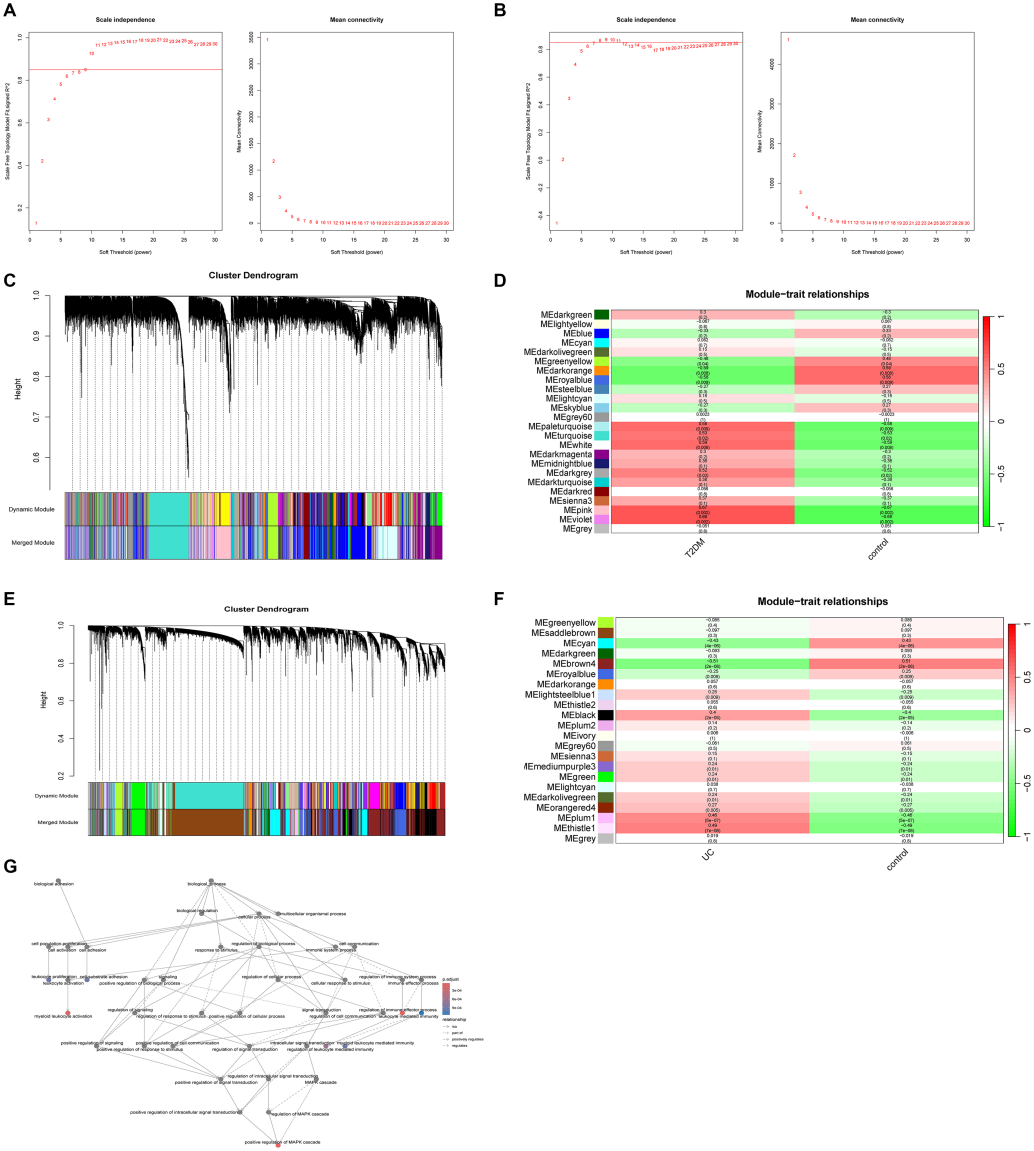
Weighted gene co-expression networks. **(A)** Scale independence and average connectivity in GSE20966. **(B)** Scale independence and average connectivity in GSE75214. **(C)** Different modules obtained from GSE20966 are displayed in various colors, aggregating genes of high relevance within each module. **(D)** Correlation analysis between modules and T2DM. **(E)** Different modules obtained from GSE75214 are displayed in various colors, aggregating genes of high relevance within each module. **(F)** Correlation analysis between modules and UC. **(G)** Biological process analysis of T2DM and UC module intercourse genes.

WGCNA analysis revealed that in T2DM, higher independence and greater biological significance were observed at β = 9. Similarly, for UC, the optimal fit was achieved at β = 8 (Figures 2A,B). When reaching the optimal fit, a hierarchical clustering dendrogram was generated, allowing the classification of similar gene expressions into distinct modules. The expression profiles within each module were then summarized using modular eigengenes (MEs), and correlations between MEs and clinical features were subsequently calculated. In T2DM, a total of 24 modules were identified, with each color denoting a distinct module. Heat maps illustrating module-trait relationships were constructed based on Spearman correlation coefficients to evaluate the association between each module and the disease (Figures 2C,D, and Supplementary Table S1). In the heat map depicting module-trait relationships, cyan signifies a negative correlation, red indicates a positive correlation, and white denotes no correlation. Six modules, namely pale turquoise, turquoise, white, dark gray, pink, and violet, exhibit substantial positive correlations with T2DM, thus qualifying them as T2DM positively correlated modules (pale turquoise module: *r* = 0.58, *p* = 0.009; turquoise module: *r* = 0.53, *p* = 0.02; white module: *r* = 0.59, *p* = 0.008, dark gray module: *r* = 0.52, p = 0.02, pink module: *r* = 0.67, *p* = 0.002, violet module: *r* = 0.66, p = 0.002). Likewise, in UC, 22 modules were identified, among which lightsteelblue1, black, mediumpurple3, green, darkolivegreen, orange4, plum1, and thistle1 exhibited positive correlations with UC (lightsteelblue1 module: *r* = 0.25, *p* = 0.009; black module: *r* = 0.40, *p* = 2e-05; mediumpurple3 module: *r* = 0.24, *p* = 0.01, green module: *r* = 0.24, *p* = 0.01, darkolivegreen module: *r* = 0.24, *p* = 0.01, orange4 module: *r* = 0.27, *p* = 0.005, plum1 module: *r* = 0.46, *p* = 5e-07, thistle1 module: *r* = 0.49, *p* = 7e-08) (Figures 2E,F, and Supplementary Table S2). Biological process analysis indicates that the interacting genes are primarily engaged in regulating the immune system and activating immune cells, among other functions (Figure 2G).

### 3.2 Identification of DEGs

**Figure 3:**
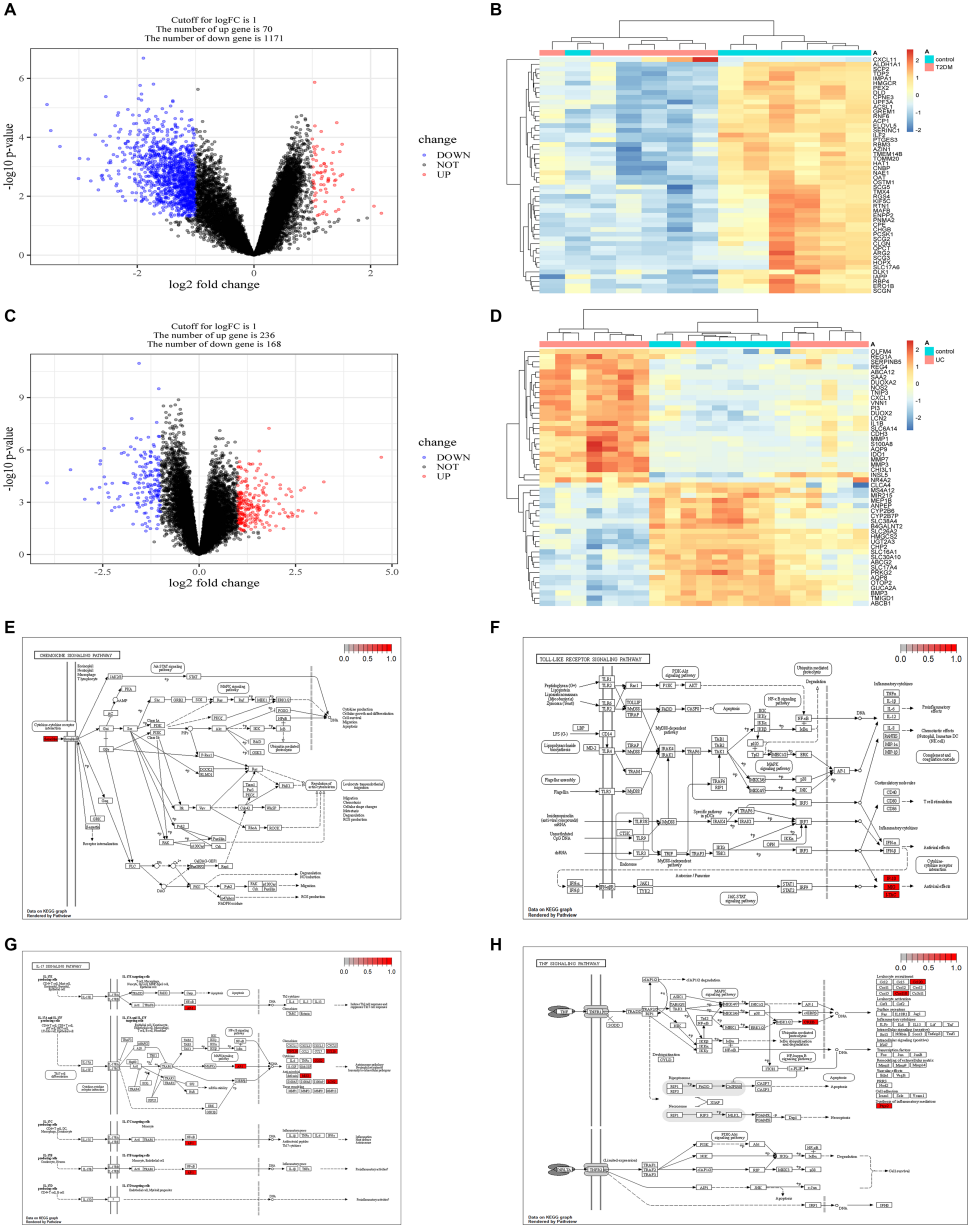
Acquisition of DEGs in T2DM and UC and KEGG enrichment analysis of genes intersecting both DEGs. **(A)** Volcano plot depicting the DEGs associated with T2DM (GSE25724). **(B)** Heatmap illustrating the DEGs associated with T2DM (GSE25724). **(C)** Volcano plot showing the DEGs associated with UC (GSE48958). **(D)** Heatmap displaying the DEGs associated with UC (GSE48958). **(E)** Chemokine signaling pathway. **(F)** Toll-like receptor signaling pathway. **(G)** IL-17 signaling pathway. **(H)** TNF signaling pathway. DEGs, Differentially Expressed Genes.

With the limma package, we identified 70 genes exhibiting high expression levels and 1,171 genes showing low expression levels associated with T2DM. Similarly, in UC, 236 genes were found to be highly expressed, while 168 genes exhibited low expression levels (Figures 3A–D, and Supplementary Tables S3, S4). The KEGG enrichment analysis of overlapping genes predominantly focused on pathways involving IL-17, TNF, Chemokine, and Toll-like receptor signaling pathways, indicating that the shared mechanism between T2DM and UC may be linked to immunity and inflammation (Figures 3E–H).

### 3.3 Immune infiltration analysis

**Figure 4:**
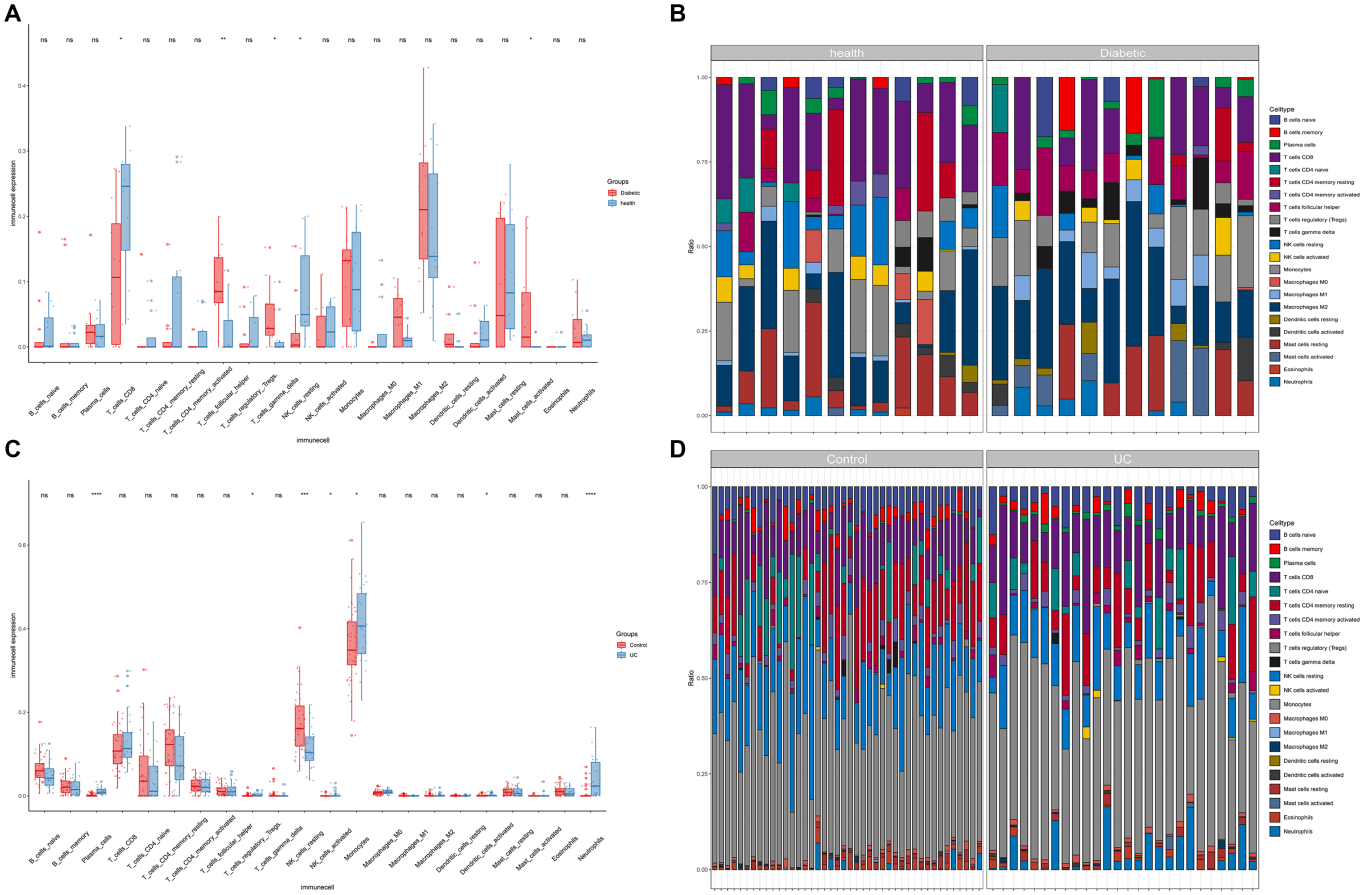
Immune infiltration analysis. **(A)** Boxplots for T2DM immune infiltration analysis. **(B)** Bar graph for T2DM immune infiltration analysis. **(C)** Boxplots for UC immune infiltration analysis. **(D)** Bar graph for UC immune infiltration analysis (*p < 0.05, **p < 0.01, ***p < 0.001).

The findings indicate a close association between the pathogenesis of T2DM and UC with the immune system (Figures 4A–D). In the T2DM group, there were observed differences in both the T cell population and resting NK cells compared to the normal group (*p* < 0.05). Compared to the normal group, significant differences (*p* < 0.05) were observed in Plasma cells, T cells regulatory (Tregs), NK cells resting, Neutrophils, NK cells activated, Monocytes, and Dendritic cells activated in UC.

### 3.4 Predictive hub genes for GQD treatment

**Figure 5:**
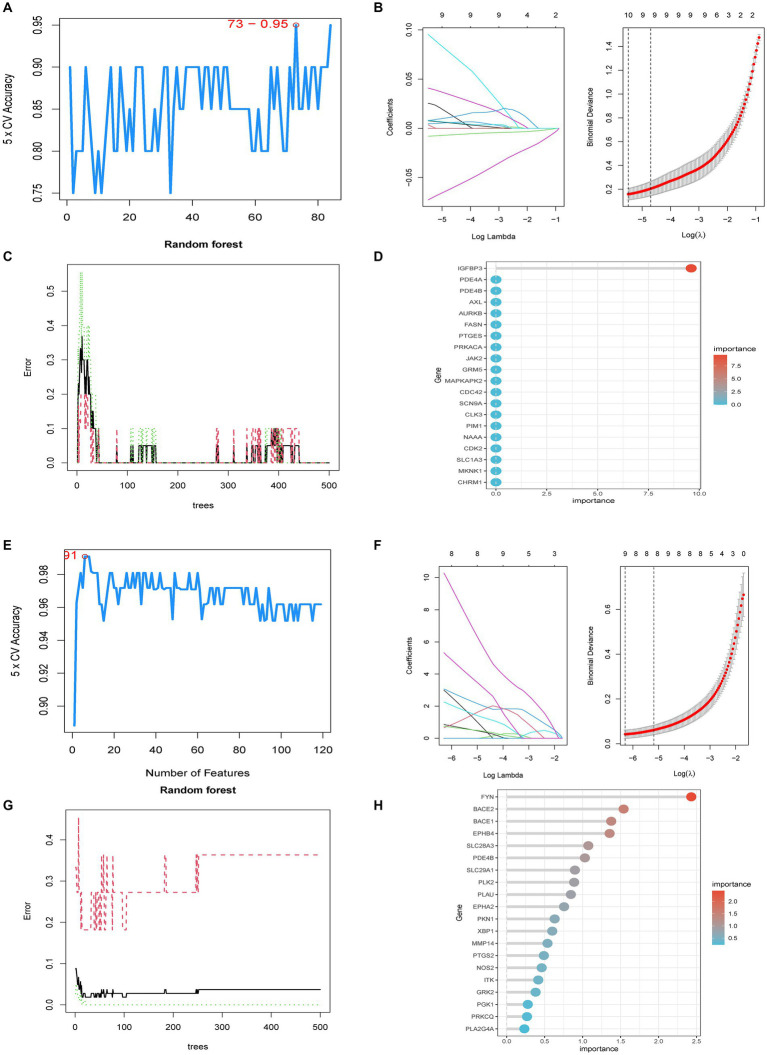

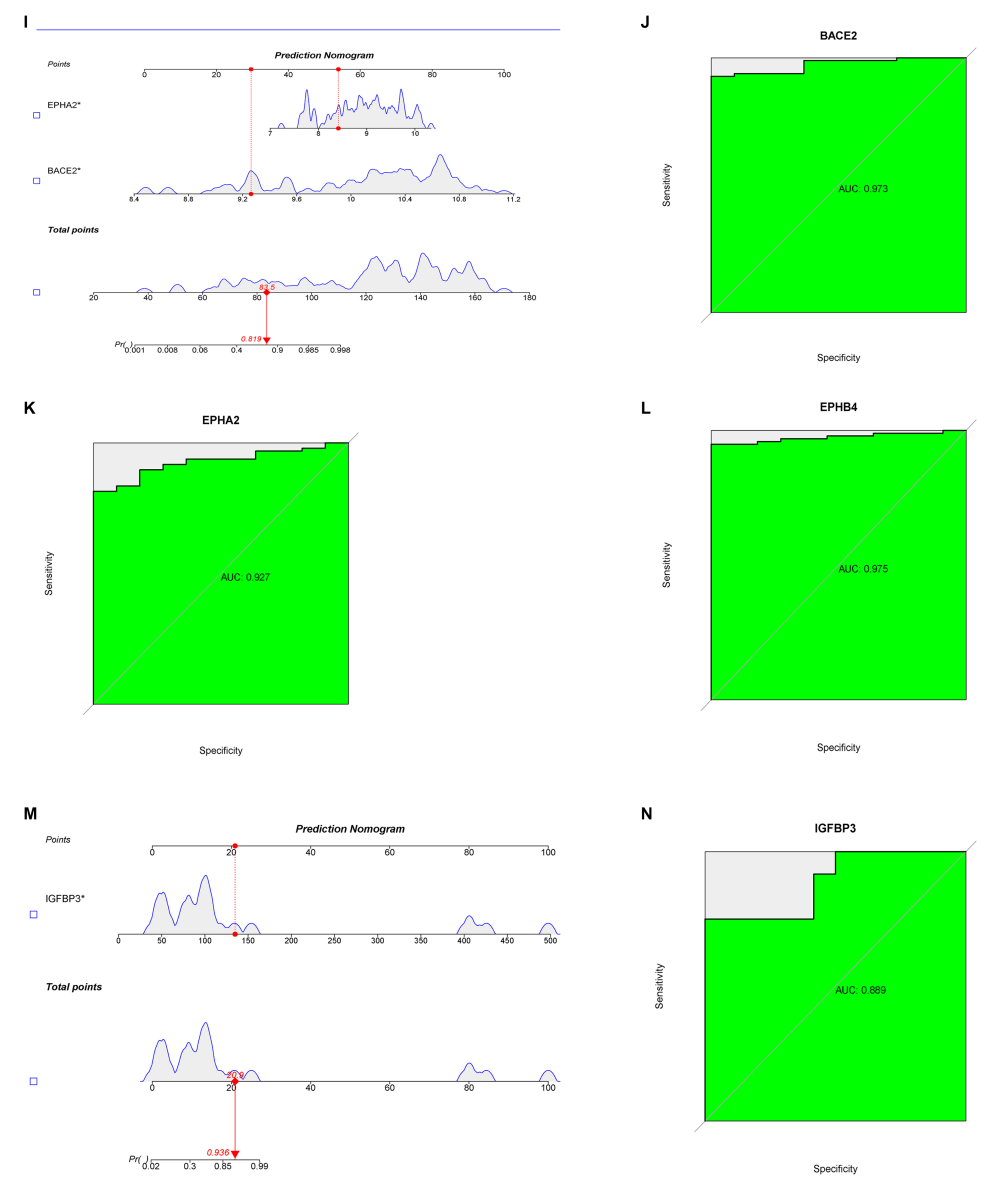
Predictive Biomarkers for GQD Treatment. **(A)** SVM-REF analysis of T2DM. **(B)** LASSO analysis of T2DM. **(C,D)** Random Forest analysis of T2DM. **(E)** SVM-REF analysis of UC. **(F)** LASSO analysis of UC. **(G,H)** Random Forest analysis of UC. **(I)** Nomograms of UC marker genes. **(J–L)** ROC curves for UC marker genes. **(M)** Nomograms of T2DM marker genes. **(N)** ROC curves for T2DM marker genes.

Potential targets for 37 core chemicals were identified through PharmMapper and SwissTargetPrediction. Afterward, the obtained results underwent the removal of identical values, culminating in a total of 444 targets (Supplementary Tables S5, S6). When employing the SVM-RFE method, we conducted 10-fold cross-validation. In the identification of core targets of GQD for treating T2DM, SVM-RFE achieved the highest accuracy of 95% with 73 features. LASSO identified 10 core targets, while RF identified one target. The intersection of core targets identified by the three models resulted in one core therapeutic target, IGFBP3 (Figures 5A–D). Applying a similar approach to identify core targets for GQD treatment of UC, SVM-RFE identified 9 core targets, LASSO also identified 9 core targets, and RF identified 37 core targets. The final intersection yielded 3 core targets: BACE2, EPHB4, and EPHA2 (Figures 5E–H). Nomograms and ROC curves depict the robust diagnostic potential of pivotal genes for both T2DM and UC (Figures 5I–N).

### 3.5 Common targets of GQD for the treatment of T2DM and UC

From the DrugBank database, we retrieved 150 targets associated with T2DM and 66 targets associated with UC. Additionally, GeneCard yielded 17,916 targets for T2DM and 5,282 targets for UC, while DisGeNET provided 2,359 targets for T2DM and 1,458 targets for UC. Furthermore, TTD identified 88 targets for T2DM and 48 targets for UC. After performing the process of removing duplicates, taking targets that appear at least twice in five databases—DrugBank, GeneCards, TTD, DisGeNET, and DEGs—and then intersecting them with the GQD target as a common target for GQD treatment of T2DM and UC. Subsequently, a total of 97 potential common targets were finalized (Supplementary Table S7).

### 3.6 PPI network

**Figure 6:**
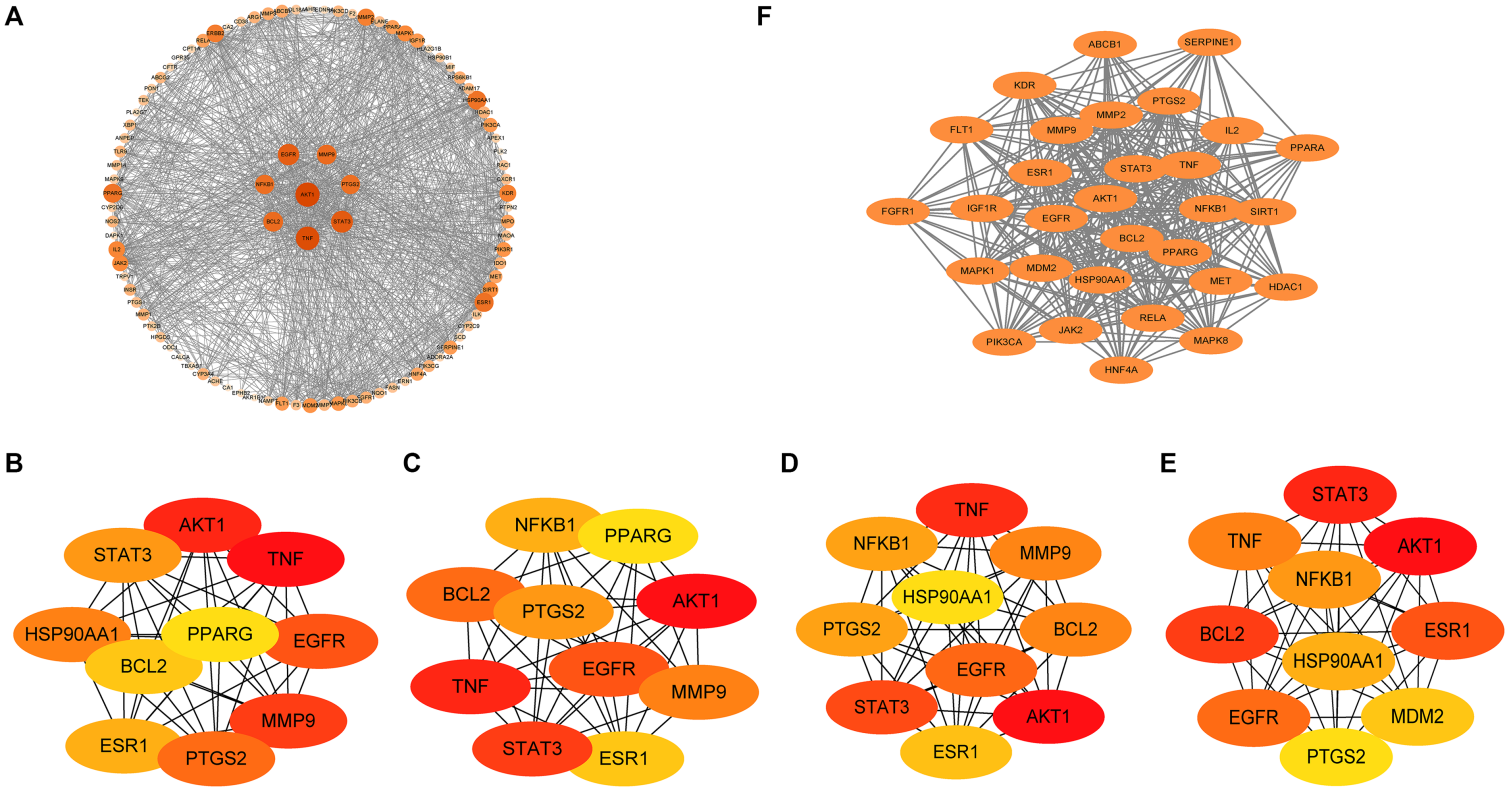
Protein-Protein interaction (PPI) network. **(A)** Analysis results of PPI network. **(B)** Betweenness centrality. **(C)** Closeness centrality. **(D)** Degree centrality. **(E)** Neighborhood Component Analysis. **(F)** MCODE plugin cluster analysis.

For the identification of common core targets of GQD for the treatment of T2DM and UC, we conducted an in-depth analysis of 97 targets using Cytoscape (Figure 6A). By considering the genes identified in the overlapping sections of the four algorithms, including DC, BC, CC, and NCC, we ultimately identified seven core target proteins of GQD for managing T2DM concomitant with UC (Figures 6B–E). The application of the MCODE plugin in cluster analysis generated highly connected sub-networks. The highest-scoring network comprised a total of 30 targets, among which the seven core targets identified previously were also encompassed (Figure 6F).

### 3.7 GO, KEGG enrichment analysis

**Figure 7:**
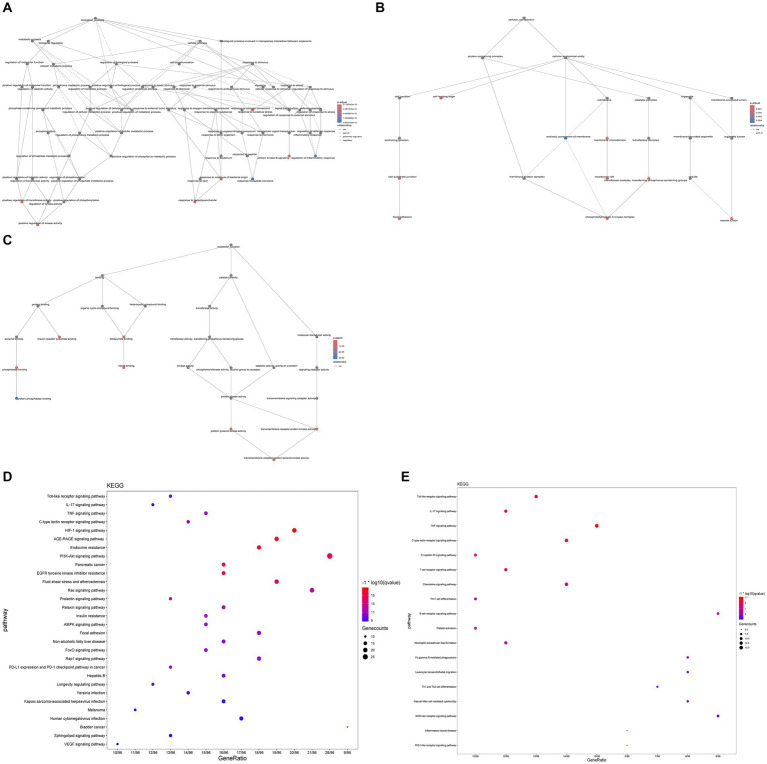
GO, KEGG enrichment analysis. **(A)** Biological process. **(B)** Cellular composition. **(C)** Molecular function. **(D)** Bubble plots of the first 30 pathways analyzed by KEGG enrichment. **(E)** KEGG enrichment analysis of immune-related pathways.

GO enrichment analysis revealed significant enrichment (*p*-value ≤0.05, *q*-value <0.01) in 1784 biological processes (BP), 105 molecular functions (MF), and 50 cellular components (CC) (Supplementary Table S8). Among these, BP mainly encompasses immune-inflammatory responses, oxidative stress, etc.; CC mainly involves membrane raft, and MF mainly includes tyrosine kinase activity, insulin receptor substrate binding, phosphatase binding, heme binding, etc. (Figures 7A–C). The KEGG enrichment analysis of potential therapeutic targets for GQD revealed pathways related to immunoinflammation, among others, indicating a broader spectrum of pathways beyond just immunoinflammatory regulation (Supplementary Table S9). We visualize the top 30 results, as well as results specifically related to immunization (Figures 7D,E).

### 3.8 Molecular docking

**Figure 8:**
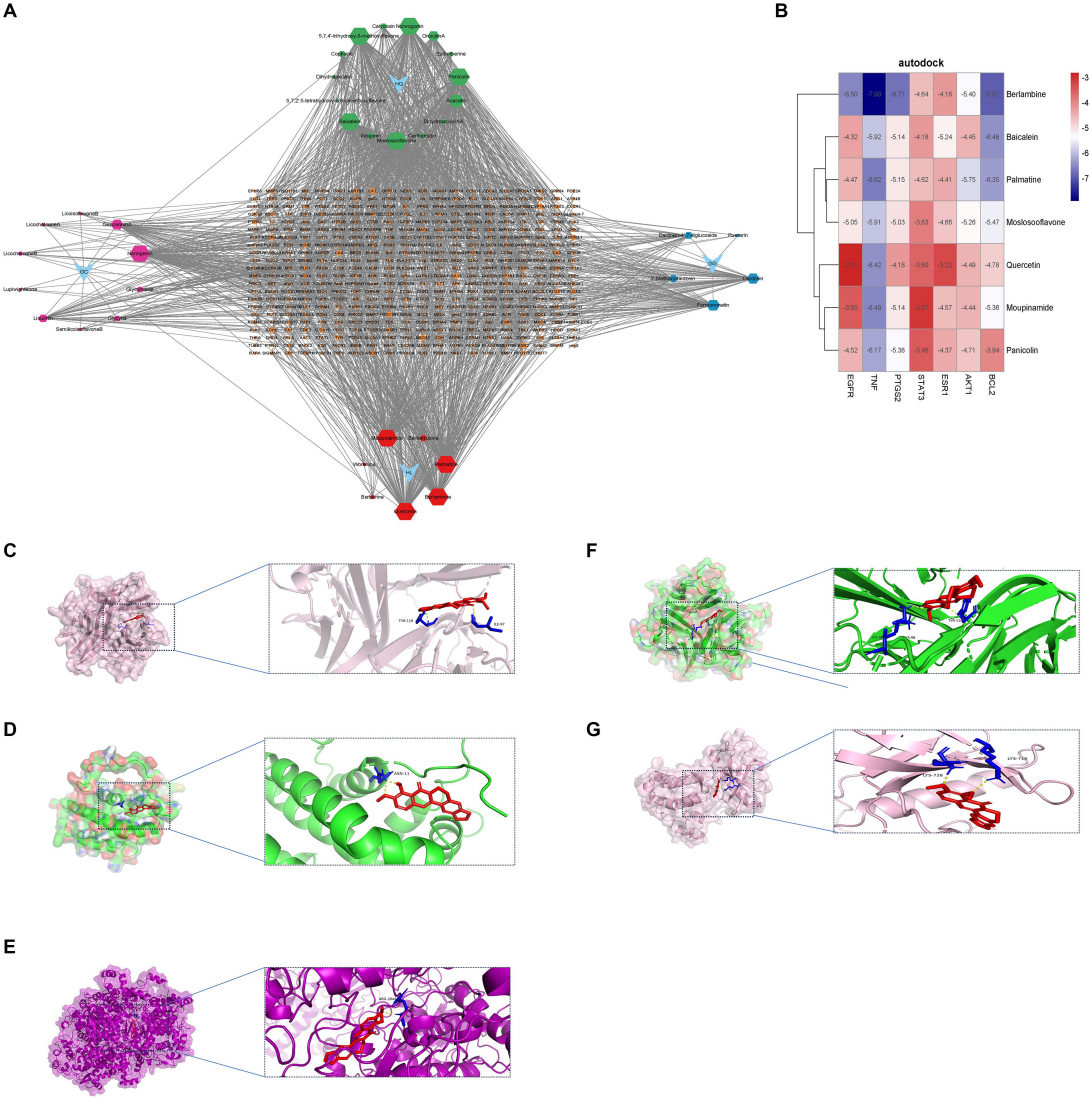
Molecular docking results. **(A)** Drug-constituent-target network diagram (the larger the value of degree in the diagram, the larger the node). **(B)** Heat map of molecular docking (kcal/mol). Berlambine – TNF **(C)**, Berlambine – BCL2 **(D)**, Berlambine – PTGS2 **(E)**, Palmatine – TNF **(F)**, Berlambine – EGFR **(G)**.

To validate our findings, we assessed the interactions between the identified active drugs and targets through molecular docking analysis. Our PPI network analysis revealed 7 core targets (AKT1, BCL2, EGFR, ESR1, PTGS2, STAT3, and TNF). Additionally, through drug-component-target network mapping, we identified 7 core chemical components of GQD: Berlambine, Palmatine, Moslosooflavone, Quercetin, Moupinamide, Panicolin, and Baicalein. Before docking, we transformed the core components and targets into the required format (Figure 8A and Table 2). The outcomes of the 49 docking combinations are represented through heatmaps and tables, highlighting the top 5 combinations exhibiting the strongest binding energy, which are then visualized in greater detail (Figures 8B–G and Table 3). In our molecular docking findings, it’s evident that Berlambine and Palmatine exhibit the strongest binding affinity to the core target.

**Table 2:** Proteins and chemicals information.

Molecule name	PubChem ID	Target	PDB ID
Berlambine	11066	EGFR	2ITV
Palmatine	19009	TNF	7KP9
Moslosooflavone	188316	PTGS2	1PXX
Quercetin	5280343	STAT3	6NJS
Moupinamide	5280537	ESR1	4XI3
Panicolin	5320399	AKT1	5AAR
Baicalein	5281605	BCL2	1G5M

**Table 3:** Binding energy.

	EGFR	TNF	PTGS2	STAT3	ESR1	AKT1	BCL2
Berlambine	−6.5	−7.9	−6.71	−4.64	−4.16	−5.4	−6.97
Palmatine	−4.47	−6.62	−5.15	−4.62	−4.41	−5.75	−6.35
Moslosooflavone	−5.05	−5.91	−5.03	−3.83	−4.66	−5.26	−5.47
Quercetin	−2.81	−6.42	−4.18	−3.6	−3.22	−4.49	−4.78
Moupinamide	−3.5	−6.49	−5.14	−3.07	−4.57	−4.44	−5.36
Panicolin	−4.52	−6.17	−5.38	−3.48	−4.37	−4.71	−3.94
Baicalein	−4.32	−5.92	−5.14	−4.18	−5.24	−4.45	−6.46

### 3.9 Single-cell sequencing analysis

**Figure 9:**
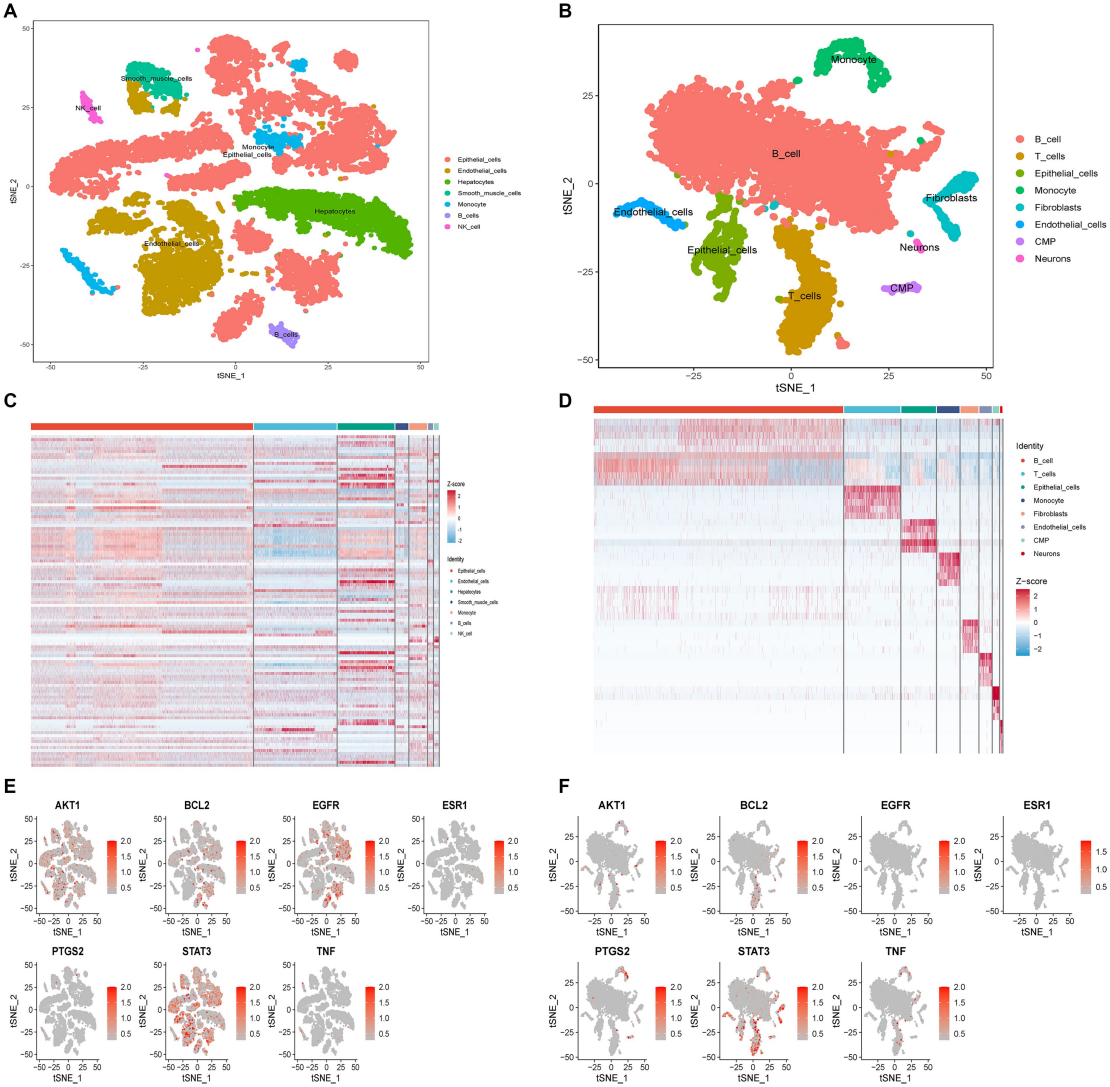
Single-cell sequencing analysis. **(A)** Cellular subtypes of T2DM. **(B)** Cellular subtypes of UC. **(C)** GQD expression in various cell clusters of T2DM. **(D)** GQD expression in various cell clusters of UC. **(E,F)** Distribution of the seven core targets in cell clusters of T2DM and UC.

The tSNE algorithm was utilized to cluster cells based on the GSE220939 and GSE231993 datasets, and subsequent labeling of each cluster was performed using SingleR. All cells from patients with T2DM were classified into seven groups: Epithelial Cells, Endothelial cells, Hepatocytes, Smooth Muscle Cells, Monocytes, B cells, and Natural Killer (NK) Cells (Figure 9A). Similarly, in UC, all cells were classified into eight classes: B cells, T cells, Epithelial cells, Monocytes, Fibroblasts, Endothelial cells, CMP, and Neurons (Figure 9B). The distribution of drug targets revealed that in T2DM, the primary cellular cluster targeted by GQD was Epithelial Cells, with subsequent impact on Hepatocytes (Figure 9C). In ulcerative colitis, GQD predominantly targets B cells, with subsequent involvement of T cells (Figure 9D). The seven core targets exhibit a broad distribution across various cell clusters (Figures 9E,F).

## 4 Discussion

An increasing body of research is corroborating the association between UC and T2DM. It has been demonstrated that diabetes is the most prevalent co-morbidity of UC (17). A population-based cohort study reveals a significantly heightened risk of T2DM among individuals with UC (18). The association between diabetes and UC holds significant implications across epidemiology, etiology, clinical practice, and therapeutic strategies, signaling profound implications for research and patient care alike (19). Hence, investigating the mechanisms underlying the co-occurrence of UC and T2DM holds clinical significance, aiding in early disease detection and timely intervention. In this study, we conducted analyses on four microarray datasets related to UC and T2DM using diverse bioinformatics approaches. Based on our predictions, inflammatory and immune processes, along with Immunoinflammatory signaling pathways such as IL-17, TNF, chemokine, and Toll-like receptor, may represent potential mechanisms underlying the co-morbidity of T2DM with UC. Following subsequent network pharmacological analyses, pivotal targets of GQD for the concurrent treatment of T2DM and UC were identified, including AKT1, BCL2, EGFR, ESR1, PTGS2, STAT3, and TNF. Notably, GQD predominantly acts on immuno-inflammatory pathways, such as Toll-like receptors, IL-17, TNF, MAPK, and the PI3K-Akt signaling pathways, in the simultaneous treatment of T2DM and UC.

In recent years, the roles of intestinal flora, inflammation, and immune regulation in the pathogenesis of T2DM and UC have attracted increasing attention (20, 21). Single-cell sequencing coupled with immune infiltration analysis underscored the pivotal role of immune cells in driving the pathogenesis of both UC and T2DM. The intestinal mucosal immune system, comprising lymph nodes, lamina propria, and epithelial cells, serves as a vital barrier safeguarding intestinal integrity. The symbiotic relationship between the microbiome and the intestinal immune system is crucial for preserving mucosal homeostasis (22). However, deficiencies and dysbiosis in the intestinal flora can result in significant impairments to the intestinal mucosal immune system, precipitating the onset of T2DM alongside UC (23, 24). UC’s development involves various factors within the gut microbiome, immune system dysfunctions, and compromised intestinal barriers, resulting in abnormal immune reactions to typical gut bacteria (25). UC is characterized by an imbalance between intestinal effector T cells and mucosal Treg, with effector T cells being overly active and Treg cells not expanding sufficiently (26). Balancing the population of Th17 and Treg cells in the intestines of mice markedly improves symptoms and reduces pathological damage in ulcerative colitis (27). In T2DM, disruptions in intestinal immunity and barrier function, alongside alterations in gut microbiota, foster heightened intestinal permeability. Consequently, intestinal bacterial components infiltrate circulation, fueling both local and systemic chronic inflammation, ultimately contributing to insulin resistance (28, 29). Dendritic cells, functioning as autocrine or paracrine modulators, synthesize and release classical neurotransmitters crucial for maintaining intestinal immune balance. Their abundance is markedly elevated in the gut of patients with UC and T2DM (30, 31). In ulcerative colitis, the usual equilibrium of intestinal B-cell reactions is disturbed, resulting in a notable decrease in regulatory B cells (32, 33). In parallel, B cells modulate Th17 proliferation and the production of pro-inflammatory factors in the intestines of T2DM patients (34). Research indicates that managing macrophage metabolism and polarization can alleviate symptoms in DSS-induced UC mice, hinting at the potential of targeting macrophage polarization to restore immune balance as a promising UC treatment strategy (35). In individuals with T2DM, there is a reduction in the quantity of anti-inflammatory T-cell subsets, such as regulatory T-cells (Treg), M2-like macrophages, and IgM-producing B-1 cells, alongside an elevation in the number and/or ratio of inflammatory effector T-cells (36). Individuals diagnosed with T2DM often exhibit irregularities in the frequency and functionality of B cells, potentially resulting in heightened inflammatory reactions and reduced insulin sensitivity. Moreover, the antibodies generated by B cells are pivotal in the progression of T2DM, notably contributing to neuroinflammation and cognitive deterioration (37, 38). In individuals with T2DM, dendritic cells are implicated in vascular dysfunction. Research indicates an elevated accumulation of dendritic cells in the perivascular adipose tissue of diabetic mice, which consequently compromises their anticonstrictive and vasodilatory functions (39). Likewise, macrophages emerge as the primary immune cell driving inflammation within pancreatic islets in T2DM, posing a threat to the insulin-secreting function of β-cells through multiple mechanisms (40). In conclusion, the elevated permeability of intestinal mucosa caused by disturbances in intestinal flora and impairment of the intestinal mucosal immune system contributes to the onset of systemic chronic inflammatory responses, a shared mechanism underlying the development of UC and T2DM (41).

Ulcerative colitis manifests as recurring mucosal inflammation with periods of remission, necessitating treatment to induce and sustain remission (42). Concurrently, the incidence of T2DM is on the rise, contributing to escalating rates of disability and mortality, thereby compounding the burden on families (43). Thus, there is an imperative to discover additional routine serum biomarkers for the early diagnosis and treatment of T2DM and UC. Three distinct machine learning studies identified IGFBP3 as a biomarker for GQD in treating T2DM, while BACE2, EPHB4, and EPHA2 emerged as biomarkers for GQD in UC treatment. IGFBP3 interacts with cellular proteins involved in glucose metabolism regulation, consequently inducing insulin resistance and diminishing glucose uptake in adipose tissue (44). For every one-unit rise in genetically determined IGFBP3 levels, there’s a 26 percent higher likelihood of developing T2DM (45). The degradation of pancreatic β-cells is a pivotal aspect of T2DM, and IGFBP3 signaling contributes to this decline in β-cell function and viability. Suppressing IGFBP3 activity can protect β-cells, potentially delaying or preventing the onset of diabetes, making it a promising therapeutic avenue for diabetes treatment (46). BACE2, a protease regulated by the JAK2/STAT5 signaling pathway, emerges as a pivotal contributor to UC development (47). The activity of IL-1R2, linked to ulcerative colitis, is influenced by the BACE2 gene. Therefore, BACE2 assumes a significant role in the pathogenesis of UC (48). The EphB/ephrin-B system has become a promising focus for tackling gut inflammatory diseases. Suppressing this system seems to provide a therapeutic benefit by regulating immune responses (49). Eph/ephrin proteins are implicated in numerous chronic inflammatory conditions. Targeting EPHB4 to disrupt EphB/ephrin B signaling holds potential as a pharmacological strategy for treating UC (50). In summary, the involvement of Eph/ephrin signaling in maintaining intestinal balance, managing inflammation, and regulating neuroimmune interactions offers exciting possibilities for future investigations and therapeutic advancements in gastrointestinal conditions (51).

In China, GQD is extensively employed for the treatment of both T2DM and UC. Through degree-value analysis of the herbal-chemical-target-protein network, we pinpointed seven active ingredients—Berlamine, Palmatine, Moslosooflavone, Quercetin, Moupinamide, Panicolin, and Baicalein—as potential compounds for treating the combined condition of T2DM and UC. Berlambine accomplishes the alleviation of inflammatory response and intestinal epithelial barrier dysfunction by diminishing the protein levels of TLR4 and MyD88, inhibiting the phosphorylation of I-κB α, and obstructing the translocation of NF-κB p65 from the cytoplasm to the nucleus (52). Concurrently, Berlambine notably increased the mRNA expression of the Nrf2 signaling pathway and elevated the activity of the pancreatic PI3K/Akt signaling pathway (53). Palmatine, a naturally occurring compound known for its anti-inflammatory and antioxidant properties, reverses the dysfunction in the insulin signaling pathway by increasing the expression of IRS-1, PI3K, AKT2, and GLUT4 genes while decreasing the expression of PKC (54). Furthermore, Palmatine alleviates ulcerative colitis symptoms by mitigating colon damage, preserving intestinal flora balance, and modulating tryptophan catabolism (55). Moslosooflavone markedly decreased the concentrations of inflammatory mediators like TNF-α, IL-1β, and IL-6 in mice (56). Quercetin’s renowned anti-inflammatory properties position it as a promising natural remedy for various inflammatory conditions (57). Quercetin ameliorates UC by restoring intestinal barrier function via the activation of AHR-mediated enhancement of tight junctions (58). Additionally, quercetin provides therapeutic benefits in T2DM by inhibiting pancreatic iron accumulation and pancreatic β-cell death (59). Panicolin exhibited strong anti-inflammatory properties by significantly suppressing the production of IL-6 induced by LPS (60). Baicalein demonstrates anti-inflammatory properties by inhibiting T cell activation and suppressing the thioredoxin system to restrict NF-κB-dependent inflammatory responses (61). Moreover, baicalein exhibits multifaceted effects on glucose metabolism, enhancing glucose uptake and glycolysis while inhibiting hepatocyte gluconeogenesis through modulation of the InsR/IRS-1/PI3K/AKT pathway (62). Simultaneously, it enhances the integrity of the intestinal epithelial barrier via the AhR/IL-22 pathway in ILC3, thereby ameliorating ulcerative colitis (63). The therapeutic efficacy of the active constituents within the herbal formulation GQD for both T2DM and UC has been substantiated.

Ultimately, the affinity between seven key target proteins and active compounds was assessed through molecular docking techniques. Berlambine and Palmatine exhibited promising binding activity to the target, implying their potential relevance to the therapeutic role of GQD in treating T2DM and UC.

It is worth noting that our study also has some limitations. At the outset, our dataset originates from various public databases, each with its own set of inclusion criteria. These distinctions could potentially impact the precision of our findings. Secondly, the sample size in the GEO database is relatively small, potentially contributing to some degree of error. Additionally, variations in algorithms and parameter selections could yield divergent outcomes and interpretations. Hence, although employing various bioinformatics and machine learning approaches, validating the results through clinical trials and animal studies is imperative.

## 5 Conclusion

In summary, we delineated potential co-morbid mechanisms between T2DM and UC, primarily implicating pathways such as IL-17, TNF, chemokine, and Toll-like receptor signaling, alongside the involvement of immune-inflammatory pathways and various immune cells like T cells, B cells, and neutrophils. Three distinct machine learning studies identified IGFBP3 as a biomarker for GQD in treating T2DM, while BACE2, EPHB4, and EPHA2 emerged as biomarkers for GQD in UC treatment. Ultimately, our investigation identified Berlambine and Palmatine as key components of GQD, presenting promising therapeutic prospects for managing the concurrent occurrence of T2DM and UC. Additionally, our study clarifies the mechanisms underlying the therapeutic effects of GQD, employing strategies that involve multiple components, targets, and pathways. This highlights its capacity to regulate immune responses and inflammation, with a specific focus on targeting toll-like receptors, IL-17, TNF, MAPK, and PI3K-Akt signaling pathways. The therapeutic strategy involving multiple components, targets, and pathways plays a vital and effective role in enhancing treatment outcomes, mitigating drug resistance, customizing treatment plans, managing complications comprehensively, and minimizing therapeutic side effects. Consequently, this approach significantly benefits patients’ clinical progress and enhances their quality of life.

## Data availability statement

The original contributions presented in the study are included in the article/Supplementary material, further inquiries can be directed to the corresponding authors.

## Author contributions

FH: Data curation, Formal analysis, Software, Visualization, Writing – original draft. LX: Data curation, Formal analysis, Software, Visualization, Writing – original draft. ZL: Investigation, Validation, Writing – review & editing. LL: Investigation, Validation, Writing – review & editing. LW: Validation, Writing – review & editing. XW: Validation, Writing – review & editing. XZ: Conceptualization, Funding acquisition, Methodology, Project administration, Supervision, Writing – review & editing. YZ: Conceptualization, Funding acquisition, Methodology, Project administration, Supervision, Writing – review & editing.

## Footnotes

^1^https://www.ncbi.nlm.nih.gov/geo/

^2^https://pubchem.ncbi.nlm.nih.gov/

^3^https://go.drugbank.com/

^4^https://www.genecards.org/

^5^https://idrblab.net/ttd/

^6^https://www.disgenet.org/

## Glossary

GQD: Gegen Qinlian Decoction
T2DM: Type 2 diabetes
UC: Ulcerative colitis
DEGs: differentially expressed genes
GEO: Gene Expression Omnibus
GO: Gene Ontology
KEGG: Kyoto Encyclopedia of Genes and Genomes
PPI: Protein–protein interaction
DC: degree centrality
BC: betweenness centrality
CC: closeness centrality
TCM: traditional Chinese medicine
DL: drug-likeness
OB: Oral bioavailability
WGCNA: Weighted Gene Co-Expression Network Analysis
FBG: fasting blood glucose
UCEIS: ulcerative colitis endoscopic index of severity
TOM: Topological Overlap Matrix
LASSO: Least Absolute Shrinkage and Selection Operator
SVM-RFE: Support Vector Machine-Recursive Feature Elimination
RF: Random Forest
